# Different combinations of monensin and narasin on growth performance, carcass traits, and ruminal fermentation characteristics of finishing beef cattle

**DOI:** 10.3389/fvets.2023.1117639

**Published:** 2023-04-28

**Authors:** Marcelo Baggio, Vinícius N. Gouvêa, José Paulo R. Barroso, Alexandre A. Miszura, Arnaldo C. Limede, Letícia C. B. Soares, Marcos Vinicius C. Ferraz, Ana Carolina S. Vicente, Evandro M. Ferreira, Rodrigo S. Marques, Alexandre V. Pires

**Affiliations:** ^1^Department of Animal Nutrition and Production, School of Veterinary Medicine and Animal Science, University of São Paulo, Pirassununga, Brazil; ^2^Texas A&M AgriLife Research and Extension Center, Amarillo, TX, United States; ^3^Department of Animal Science, Texas A&M University, College Station, TX, United States; ^4^Department of Animal Science, Federal University of Amazonas, Parintins, Brazil; ^5^Department of Animal Sciences, University of Florida, Gainesville, FL, United States; ^6^Department of Animal Science, “Luiz de Queiroz” College of Agriculture, University of São Paulo, Piracicaba, Brazil; ^7^Department of Animal and Range Sciences, Montana State University, Bozeman, MT, United States

**Keywords:** adaptation, feed additives, feedlot, intake, ionophore, rumen

## Abstract

The objective of this study was to evaluate the effects of different combinations of monensin and narasin on finishing cattle. In Exp. 1, 40 rumen-cannulated Nellore steers [initial body weight (BW) = 231 ± 3.64 kg] were blocked by initial BW and assigned to one of the five treatments as follows: Control (CON): no feed additive in the basal diet during the entire feeding period; Sodium monensin (MM) at 25 mg/kg dry matter (DM) during the entire feeding period [adaptation (days 1–21) and finishing (days 22–42) periods]; Narasin (NN) at 13 mg/kg DM during the entire feeding period (adaptation and finishing periods); Sodium monensin at 25 mg/kg DM during the adaptation period and narasin at 13 mg/kg DM during the finishing period (MN); and narasin at 13 mg/kg DM during the adaptation period and sodium monensin at 25 mg/kg DM during the finishing period (NM). Steers fed MM had lower dry matter intake (DMI) during the adaptation period compared to NM (*P* = 0.02) but not compared to CON, MM, MN, or NN (*P* ≥ 0.12). No differences in DMI were observed among the treatments during the finishing (*P* = 0.45) or the total feeding period (*P* = 0.15). Treatments did not affect the nutrient intake (*P* ≥ 0.51) or the total apparent digestibility of nutrients (*P* ≥ 0.22). In Exp. 2, 120 Nellore bulls (initial BW = 425 ± 5.4 kg) were used to evaluate the effects of the same treatments of Exp. 1 on growth performance and carcass characteristics of finishing feedlot cattle. Steers fed NM had greater DMI during the adaptation period compared to CON, MM, and MN (*P* ≤ 0.03), but no differences were observed between NM and NN (*P* = 0.66) or between CON, MM, and NN (*P* ≥ 0.11). No other differences between treatments were observed (*P* ≥ 12). Feeding narasin at 13 mg/kg DM during the adaptation period increases the DMI compared to monensin at 25 mg/kg DM, but the feed additives evaluated herein did not affect the total tract apparent digestibility of nutrients, growth performance, or carcass characteristics of finishing cattle.

## 1. Introduction

According to Brown et al. (1) and Pereira et al. (2), the adaptation period in which finishing cattle is transitioned from a high-roughage-based diet to a high-concentrate diet is considered the most critical period for feedlot cattle due to the changes in dry matter intake (DMI) and risks of subacute or acute acidosis caused by the increased amount of rapidly fermentable carbohydrates in the diet (3–5). The low DMI during the first 14–21 days of the adaptation period (2, 4, 6) can limit the performance of feedlot cattle. Therefore, feed additives that can modulate ruminal fermentation characteristics and increase the average daily gain (ADG) without negatively affecting the DMI would benefit the adaptation process and perhaps increase growth performance throughout the feedlot finishing phase.

Ionophores, such as sodium monensin, are largely used in the feedlot industry to improve the feed efficiency of finishing cattle. According to the surveys conducted by Samuelson et al. (7) and Pinto and Millen (8), more than 97% of feedlot nutritionists in the USA and Brazil include ionophores in finishing diets for feedlot cattle. However, according to Samuelson et al. (7), monensin is the primary ionophore included in feedlot diets in the USA. Monensin decreases DMI, especially in diets with a high proportion of forage (9). In a meta-analysis conducted by Duffield et al. (10), monensin decreased DMI by 3% and improved feed efficiency (G:F; gain to feed ratio) of finishing beef cattle by 2.5–3.5%. The positive effect of feeding monensin to ruminants is frequently attributed to the improved efficiency of energy metabolism as a result of increased propionate production in the rumen (11). Monensin can also affect meal patterns, especially during times when rumen pH is low (12). According to Erickson et al. (13), feeding monensin to finishing feedlot cattle increases the number of meals and decreases intake rate (%/h) and average meal size. However, feeding monensin reduces ruminal pH variance (13), which can contribute to preventing rumen acidosis on high-grain finishing diets (14).

Narasin is an ionophore that has been studied in high-roughage diets (15–17). According to Polizel et al. (18), feeding Narasin at 13 or 20 mg/kg DM to beef steers provided with a high-roughage diet (Tifton-85; *Cynodon dactylon* spp.) did not affect DMI and increased ruminal concentration of propionate. Feeding narasin at 13 mg/kg DM increased growth performance and benefited ruminal fermentation characteristics of steers fed a forage-based diet (19).

Therefore, we hypothesized that feeding narasin during the adaptation period would increase the DMI and growth performance compared to monensin, and the positive effects obtained during the adaptation period would carry over the entire feedlot finishing phase. The objective of this experiment was to evaluate different combinations of monensin and narasin on intake, nutrient digestibility, ruminal fermentation characteristics, growth performance, and carcass traits of finishing feedlot cattle.

## 2. Materials and methods

A total of two experiments were conducted at the Experimental Feedlot Cattle facilities of the Department of Animal Science and the “Luiz de Queiroz” College of Agriculture (ESALQ), University of São Paulo (USP), in Piracicaba, State of São Paulo, Brazil (22°43'30” S, 47°38'51” W). All procedures using animals were approved by the Animal Care and Use Committee of the ESALQ/USP (protocol number #9763030920).

### 2.1. Experiment 1. Intake, digestibility, and ruminal fermentation characteristics

**Figure 1:**
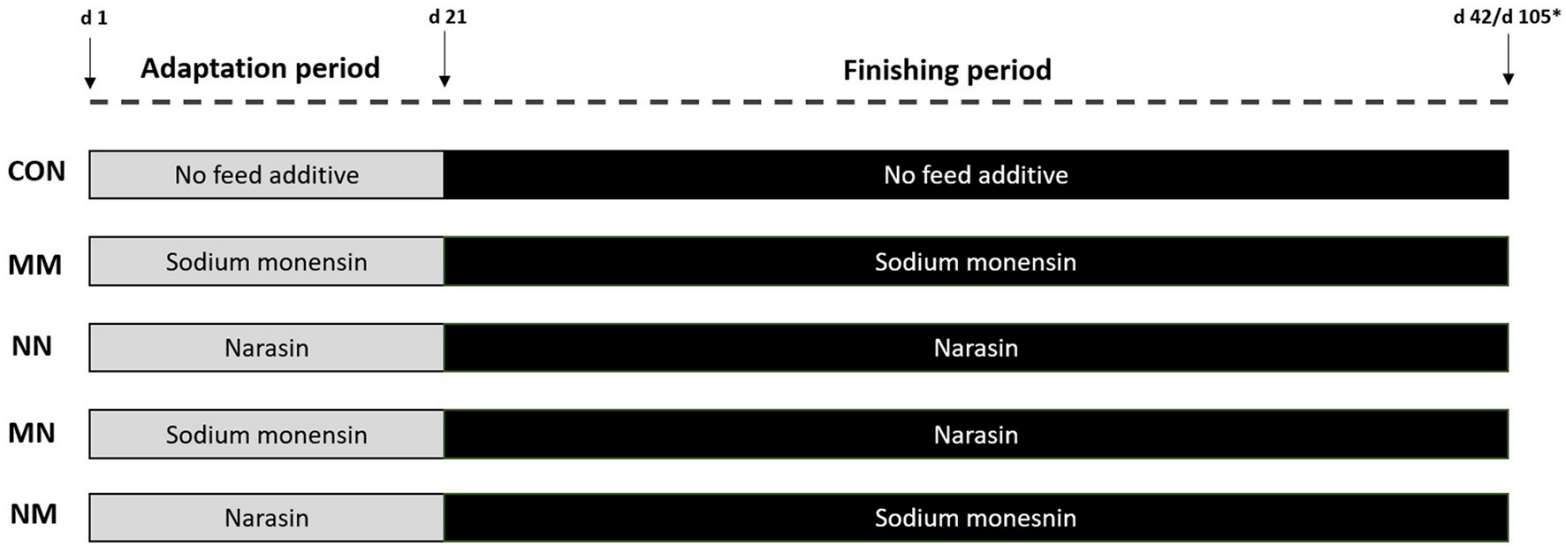
Schematic representation of the treatments. The adaptation period consisted of 21 days. During the adaptation period, steers were fed three step-up diets which gradually (7 days in each step) decreased the roughage source (sugarcane bagasse) from 23 to 18% (Adap. 1), to 13% (Adap. 2), and to 8% (Adap. 3) and increased concentrate accordingly. From days 22 to 42 (Exp. 1) or day 105 (Exp. 2), steers were fed the finishing diet containing 8% of roughage (sugarcane bagasse). Treatments were as follows: Control (CON): no feed additive in the basal diet during the entire feeding period; Sodium monensin (MM) at 25 mg/kg DM during the entire feeding period [adaptation and finishing periods (Rumensin 100, Elanco Brazil, São Paulo, SP, Brazil)]; Narasin (NN) at 13 mg/kg DM during the entire feeding period (adaptation and finishing periods; Zimprova 100, Elanco Brazil, São Paulo, SP, Brazil); Sodium monensin at 25 mg/kg DM during the adaptation period and narasin at 13 mg/kg DM during the finishing period (MN); and narasin at 13 mg/kg DM during the adaptation period and sodium monensin at 25 mg/kg DM during the finishing period (NM). *Exp. 1 lasted for 42 days and Exp. 2 lasted for 105 days.

A total of 40 rumen-cannulated Nellore steers [*Bos indicus*; initial body weight (BW) = 231 ± 3.64 kg; age = 20 ± 1.0 months] were blocked by initial BW and allocated to 40 pens (3.5 × 8 m; 1 steer/pen), with concrete floor, fully roofed, 3.5 m of bunk space, and individual waterers (BV 009 3L, Agricola Suin, Joinville, SC, Brazil). Pens within each BW block were then randomly assigned to one of the five treatments (Figure 1) as follows: (1) Control (CON): no feed additive in the basal diet during the entire feeding period; (2) Sodium monensin (MM) at 25 mg/kg dry matter (DM) during the entire feeding period [adaptation (days 1–21) and finishing (days 22–42) periods (Rumensin 100, Elanco Brazil, São Paulo, SP, Brazil)]; (3) Narasin (NN) at 13 mg/kg DM during the entire feeding period (adaptation and finishing periods; Zimprova 100, Elanco Brazil, São Paulo, SP, Brazil); (4) Sodium monensin at 25 mg/kg DM during the adaptation period and narasin at 13 mg/kg DM during the finishing period (MN); and (5) narasin at 13 mg/kg DM during the adaptation period and sodium monensin at 25 mg/kg DM during the finishing period (NM).

The experiment lasted for 42 days. Steers were adapted to the finishing diet during the first 21 days of the experiment (adaptation period; day 1–21), using three step-up diets (Table 1) which gradually (7 days in each step) decreased the roughage source (sugarcane bagasse) from 23 to 18% (Adap. 1), to 13% (Adap. 2), and to 8% (Adap. 3) and increased concentrate accordingly. The finishing diet containing 8% of roughage (DM basis) was fed from days 22 to 42 (finishing period). The experimental diets (Table 1) were formulated to meet the nutrient requirements of finishing Nellore steers for 1.35 kg ADG as specified by NASEM (9). The experimental rations were mixed using a feed-mix wagon (Totalmix TMX25, Casale Equipamentos Ltda., São Carlos, Brazil) and delivered to the steers as a total mixed ration (TMR) once a day at 800 h. After mixing, the TMR was weighed into 100 L capacity plastic bins using an electronic scale with 50 g of readability (Welmy, W 300, Santa Bárbara d'Oeste, SP, Brazil) and manually delivered to each pen. Steers had *ad libitum* access to the experimental diets containing the treatments and water during the entire experiment (days 1–42). The amount of feed offered to each steer was adjusted daily based on the amount of feed provided on the previous day to allow 5% of refusals, that were removed daily, weighted, sampled (5% of the total amount; wet weight), and frozen (−18°C) for analysis of nutrient composition and DMI calculation. Samples of ingredients (~500 g) were collected one time each week and dried at 105°C for 24 h for DM determination and diet DM adjustments.

**Table 1:** Ingredients and chemical composition [dry matter basis (DM)] of experimental diets used in Exp. 1 and 2.

	**Experimental diets** ^**a**^			
**Item**	**Adap. 1**	**Adap. 2**	**Adap. 3**	**Finishing** ^b^
Days in each diet	7	7	7	21/84
**Ingredients, %**				
Sugarcane bagasse	23.0	18.0	13.0	8.00
Whole cottonseed	10.0	10.0	10.0	10.0
Soybean hulls	15.0	15.0	15.0	15.0
Ground corn	44.0	49.0	56.0	62.0
Soybean meal	5.00	5.00	3.00	2.00
Urea	1.00	1.00	1.00	1.00
Mineral supplement^c^	2.00	2.00	2.00	2.00
**Analyzed composition, %**				
Dry matter	70.8	71.9	70.2	70.0
Organic matter	92.2	92.9	91.6	91.7
Crude protein	14.6	14.5	12.7	12.4
Neutral detergent fiber	45.4	43.4	30.5	28.0
Acid detergent fiber	26.9	25.7	17.1	16.0
Ash	7.70	7.00	8.30	8.20
Ether extract	3.30	3.20	3.10	4.40
Net energy of maintenance, Mcal/kg^d^	1.69	1.77	1.82	1.90
Net energy of gain, Mcal/kg^d^	1.07	1.15	1.23	1.30
Total digestible nutrients^d^	71.5	74.3	77.2	80.1

^a^Adap, adaptation diet; Adap. 1, fed from days 1 to 7; Adap. 2, fed from days 8 to 14; Adap. 3, fed from days 15 to 21; Finishing, finishing diet.

^b^In Exp. 1, steers were fed the finishing diet for 21 days. In Exp. 2, steers were fed the finishing diet for 84 days.

^c^Containing (DM basis): 164 g/kg Ca, 60 g/kg P, 40 g/kg S, 140 g/kg Na, 10 g/kg Mg, 780 mg/kg Mn, 3,750 mg/kg Zn, 1,010 mg/kg Cu, 60 mg/kg Co, 75 mg/kg I, and 19 mg/kg Se.

^d^Estimated using the tabular values according to NASEM (9) with no feed additive inclusion. Energy values of sugarcane bagasse were obtained from Valadares Filho et al. (20).

Ruminal content was collected from each steer 6 h after feeding on days 21 (the last day of the adaptation period), 24, 27, and 42 (end of the experiment). During the sampling days, five trained personnel were assigned to eight steers each; at least one steer of each treatment/staff, so treatments were balanced within personnel. In addition, trained personnel rotated on each sampling day to account for any variation during sampling. Steers were halter broken and used in the previous study (19) and did not require to be restrained for sampling. Sampling was completed within 40 min. Approximately 100 ml of ruminal content was manually collected from the ventral portion of the rumen and squeezed into four layers of cheesecloth as described by Polizel et al. (18). Immediately after collection, the pH of ruminal fluid was measured using a digital pH meter (Digimed-M20; Digimed Instrumentação Analítica; São Paulo, SP, Brazil). Sub-samples (~5 ml) were stored at −18°C for further analysis of short-chain fatty acids (SCFA) and ruminal ammonia nitrogen N (NH_3_-N).

The SCFA concentration was determined as described by Polizel et al. (18). In brief, 1.6 ml of ruminal fluid was mixed with 0.4 mL metaphosphoric acid:formic acid (3:1) and 0.2 mL of 100 mM 2-ethyl-butyric acid (internal standard). The homogenate was centrifuged for 30 min at 15,000 × *g* at 4°C, and 1.2 ml of the supernatant was then transferred to a chromatography vial. The quantification of SCFA was performed using an Agilent 7890A gas chromatograph equipped with a flame ionization detector (7683B), a fused-silica capillary column (J &W19091F-112, Agilent Technologies, Santa Clara, CA, United States), 25 m in length, and 320 mm internal diameter, containing 0.20 M cyanopropyl polysiloxane. The data acquisition was performed using the ChemStation software (Agilent Technologies, Santa Clara, CA, United States). The concentration of NH_3_-N was determined by the colorimetric method as described by Chaney and Marbach (21), adapted for a microplate reader (EON, BioTech Instruments, Winooski, VT, United States), using a 550 nm absorbance filter.

The total fecal collection was performed from days 37–41 to determine the total apparent digestibility of nutrients. The total fecal production of each steer was collected every 4 h from the concrete floor (22), weighted, sampled (10% of the total), and frozen at −18°C for further chemical analysis.

At the end of the experiment, feed ingredients and fecal samples were thawed, dried in a forced-air oven at 55°C for 72 h, and ground through a 1-mm screen using a Willey-type mill (MA-680, Marconi, Piracicaba, Brazil). All samples were analyzed for DM [method 930.15; (23)], ash [method 942.05; (23)], and nitrogen (Leco FP- 528; Leco Corp., St. Joseph, MI). The organic matter (OM) was calculated based on ash values (OM, % = 100 – ash, %). Crude protein (CP) was obtained by multiplying the total N content by 6.25. The determination of the fibrous fraction was carried out sequentially, using thermostable alpha-amylase and sodium sulfite for analysis of neutral detergent fiber (NDF) according to the methodology proposed by van Soest et al. (24) and acid detergent fiber (ADF) according to Goering and van Soest (25), using the Ankon 2000 Fiber Analyzer (Ankon Tech. Corp., Fairport, NY, United States). The NDF and ADF reports are ash corrected.

### 2.2. Experiment 2. Animal performance, feeding behavior, and carcass characteristics

A total of 120 Nellore bulls (*Bos indicus;* initial BW = 425 ± 5.4 kg; age = 30 ± 2.0 months) purchased from one single ranch were used in this experiment. Upon feedlot arrival, bulls were individually weighted (Id Beck 2.0, Beckhauser Balanças e Troncos, Paranavaí, Brasil), after 16 h of feed and water withdrawal, identified with ear tags, and vaccinated/dewormed as described by Gouvêa et al. (26). Bulls were blocked according to the initial BW, allocated to 40 pens as described in Exp. 1 (3.5 × 8 m; 3 bulls/pen), and then, pens within each BW block were assigned to the same treatments described in Exp. 1 (Figure 1). Experiment 2 lasted for 105 days. The experimental diets and adaptation protocol were the same as described in Exp. 1 (Table 1). The finishing diets were fed from days 22 to 105. Bulls had *ad libitum* access to water and to the diets containing the treatments throughout the experiment. Feeding management, diet, and refusal sampling were as described in Exp. 1.

Bulls were individually weighed after 16 h of fasting (feed and water) at the beginning of the experiment (day 1) and at the end of the trial (day 105). Full BW was collected at the end of the adaptation period (day 21) and discounted by 4% as ruminal fill (9) to calculate shrunk BW at the end of the adaptation period. The ADG, DMI, and G:F were calculated for each experimental period. At the end of the experiment (day 105), bulls were transported to a commercial packing plant and slaughtered on the following day as described by Gouvêa et al. (26). In brief, the hot carcass weight (HCW) was obtained after the removal of the hide, head, feet, tail, kidneys, and visceral fat. The dressing percentage was calculated using the HCW obtained after slaughter, divided by the final shrunk weight. *Longissimus muscle* (**LM**) area and subcutaneous fat thickness were measured between the 12 and 13th rib from each carcass after a 24-h chill at 2°C as described by Toseti et al. (27), using a digital camera attached to a fixed distance (10 cm) of a 15 × 20 cm rectangular steel base. The images obtained by the digital camera were interpreted by one experienced technician using the AutoCAD^®^ software.

### 2.3. Statistical analysis

Both experiments were analyzed using the MIXED procedure of SAS 9.4 software (SAS Inst. Inc., Cary, NC), as a randomized complete block design with five treatments and initial BW used as the blocking factor. The steer was the experiment unit in Exp. 1, and the pen served as the experimental unit in Exp. 2. The Kenward Roger approximation was used to determine the correct denominator degrees of freedom for testing fixed effects. Each experimental period (adaptation, finishing, and total feeding period) was analyzed separately. In Exp. 1, the statistical model used to analyze intake and nutrient digestibility was: yij = μ + Ti + Bj + eij, where y = dependent variable, μ = overall mean, Ti = fixed effect of treatment, Bi = random effect of block, and eij = the residual error. The SCFA, pH, and NH_3_-N in Exp. 1 were analyzed as repeated measurements over time using the MIXED procedure following the statistical model: yijk = μ+ Ti + Bj + eij + Pk + TiPk + eijk, where μ = overall mean, Ti = the fixed effect of treatment; Bj = random block effect, eij = subject level random error, Pk = fixed effect of time, TiPk = fixed effect of treatment × time interaction, and eijk = the residual error. The covariance matrix used was the compound symmetry (CS) and was selected using the Bayesian information fit criteria (smaller is better), after adjusting models with the AR(1), ARH(1), ANTE(1), CS, CSH, and UN covariance. The subject was treatment (pen). In Exp. 2, the statistical model used to analyze growth performance and carcass data was as follows: yij = μ + Ti + bj + eij, where μ = overall mean, Ti = fixed effect of treatments, bi = random effect of a block, and eij = the residual error.

Data from all experiments were reported as least-square means. Effects were declared significant at *P* < 0.05. The tendency was discussed when *P* > 0.05 and ≤ 0.10. When a significant treatment effect was observed, a *post-hoc* analysis using the Tukey test was used to identify significant differences among the treatment's least square means. When a significant treatment × time interaction was observed in Exp. 2 for SCFA, NH_3_-N, or pH, treatments were compared within each time point using the Tukey test.

## 3. Results

### 3.1. Experiment 1

Feed additives affected the DMI during the adaptation period (*P* = 0.02; Table 2). Steers fed MM had lower DMI during the adaptation period compared to NM (*P* = 0.02; 5.76 vs. 6.92 kg/day, respectively) but not compared to CON, MM, MN, or NN (*P* ≥ 0.12). No differences in DMI were observed between the treatments during the finishing period (*P* = 0.45) or the total feeding period (*P* = 0.15).

**Table 2:** Effects of feed additives on dry matter intake of finishing beef cattle—Exp. 1.

**Item**	**Treatments** ^**a**^					**SEM^b^**	***P-*value**
	**CON**	**MM**	**MN**	**NM**	**NN**		
Pens (steers)	8 (8)	8 (8)	8 (8)	8 (8)	8 (8)	-	-
**Dry matter intake, kg/day**							
Adaptation period (days 1–21)	6.65^cd^	5.76^d^	6.06^cd^	6.92^c^	6.57^cd^	0.26	0.02
Finishing period (days 22–42)	7.85	6.95	7.57	7.77	7.43	0.36	0.45
Total feeding period (days 1–42)	7.25	6.35	6.87	7.34	7.00	0.28	0.15

^a^CON, control; no feed additive; MM, sodium monensin at 25 mg/kg DM during the adaptation and finishing periods (Rumensin, Elanco Saude Animal, São Paulo, SP, Brazil); MN, sodium monensin at 25 mg/kg DM during the adaptation period and narasin at 13 mg/kg DM (Zimprova, Elanco Saude Animal, São Paulo, SP, Brazil) during the finishing period; NM, narasin at 13 mg/kg DM during the adaptation period and sodium monensin at 25 mg/kg DM of during the finishing period; NN, narasin at 13 mg/kg DM during the adaptation and finishing periods.

^b^SEM, standard error of the mean.

^cd^Means that do not have common superscript letters are different (Tukey's test; *P* < 0.05).

Treatments did not affect the nutrient intake (*P* ≥ 0.51) and the total tract apparent digestibility of nutrients (*P* ≥ 0.22; Table 3).

**Table 3:** Effect of feed additives on intake and apparent digestibility of nutrients of finishing beef cattle—Exp. 1; days 37–42.

**Item**	**Treatments** ^**a**^					**SEM^b^**	***P*-value**
	**CON**	**MM**	**MN**	**NM**	**NN**		
Pens (steers)	8 (8)	8 (8)	8 (8)	8 (8)	8 (8)	-	-
**Intake, kg/day**							
Dry matter	7.03	6.33	7.03	6.51	6.34	0.49	0.71
Organic matter	6.34	5.86	6.47	6.03	5.83	0.45	0.81
Neutral detergent fiber	1.90	1.74	2.08	1.80	1.92	0.14	0.51
Acid detergent fiber	1.07	1.02	1.19	1.05	1.10	0.08	0.67
**Apparent digestibility, %**							
Dry matter	67.5	66.7	68.7	67.0	63.5	1.67	0.27
Organic matter	68.9	69.2	70.3	69.6	66.0	1.57	0.35
Neutral detergent fiber	52.9	53.1	59.1	52.1	54.2	2.22	0.22
Acid detergent fiber	51.0	54.2	57.7	54.2	53.9	2.36	0.46

^a^CON, control; no feed additive; MM, sodium monensin at 25 mg/kg DM during the adaptation and finishing periods (Rumensin, Elanco Saude Animal, São Paulo, SP, Brazil); MN, sodium monensin at 25 mg/kg DM during the adaptation period and narasin at 13 mg/kg DM (Zimprova, Elanco Saude Animal, São Paulo, SP, Brazil) during the finishing period; NM, narasin at 13 mg/kg DM during the adaptation period and sodium monensin at 25 mg/kg DM of during the finishing period; NN, narasin at 13 mg/kg DM during the adaptation and finishing periods.

^b^SEM, standard error of the mean.

No treatment × day interaction was observed for any of the ruminal fermentation characteristics evaluated in the present study (*P* ≥ 0.14; Table 4). Treatments tended to affect the ruminal concentration of NH_3_-N (*P* = 0.06). Steers fed NN tended to have greater NH_3_-N concentration compared to CON (*P* = 0.15; 5.81 vs. 4.11 mg/dL, respectively) but not compared with MM, MN, or NM (*P* ≥ 0.20). No other treatment effects were observed on the ruminal fermentation characteristics (*P* ≥ 0.61).

**Table 4:** Effect of feed additives on the ruminal concentration of short-chain fatty acids (SCFAs), pH, and ammonia nitrogen (NH_3_-N) of finishing beef cattle—Exp. 1.

**Item**	**Treatments** ^**a**^					**SEM^b^**	***P*** **-value** ^**c**^		
	**CON**	**MM**	**MN**	**NM**	**NN**		**Treat**	**Day**	**Treat** × **Day**
Pens (steers)	8 (8)	8 (8)	8 (8)	8 (8)	8 (8)	-	-	-	-
Total SCFA, mmol/L	119	112	125	117	119	8.91	0.86	<0.001	0.90
**SCFA, mol/100 mol**									
Acetate	58.9	58.9	57.6	57.9	59.1	1.80	0.96	<0.001	0.34
Propionate	27.1	26.6	26.9	27.5	26.7	1.61	0.92	<0.001	0.15
Butyrate	9.77	10.8	11.7	10.7	10.7	0.71	0.35	0.06	0.22
Acetate:propionate ratio	2.31	2.31	2.24	2.27	2.48	0.06	0.92	<0.01	0.14
Ruminal pH	6.20	6.08	6.00	6.08	6.10	0.11	0.79	<0.01	0.43
NH_3_-N mg/dL	4.11^e^	5.21^de^	5.75^de^	4.11^de^	5.81^d^	0.55	0.06	0.23	0.72

^a^CON, control; no feed additive; MM, sodium monensin at 25 mg/kg DM during the adaptation and finishing periods (Rumensin, Elanco Saude Animal, São Paulo, SP, Brazil); MN, sodium monensin at 25 mg/kg DM during the adaptation period and narasin at 13 mg/kg DM (Zimprova, Elanco Saude Animal, São Paulo, SP, Brazil) during the finishing period; NM, narasin at 13 mg/kg DM during the adaptation period and sodium monensin at 25 mg/kg DM of during the finishing period; NN, narasin at 13 mg/kg DM during the adaptation and finishing periods.

^b^SEM, standard error of the mean.

^c^Treat, effect of treatment; day, effect of day (days 21, 24, 27, and 42); Treat × Day = interaction between treatment and day.^de^Means that do not have common superscript letters are different (Tukey's test; *P* < 0.05).

An effect of the day (*P* ≤ 0.01) was observed for the total concentration of SCFA, molar proportion of acetate, propionate, and ruminal pH (Tables 4, 5). The sampling day tended (*P* = 0.06) to affect the molar proportion of butyrate (Tables 4, 5). Overall, ruminal pH, molar proportion of acetate and butyrate, and acetate:propionate ratio decreased over the sampling days (*P* < 0.05), and the total ruminal concentration of SCFA and molar proportion of propionate increased (*P* < 0.05) over the sampling days (Table 5).

**Table 5:** Effect of sampling days on the ruminal concentration of short-chain fatty acids (SCFAs) and pH of finishing beef cattle—Exp. 1.

**Item**	**Sampling days** ^**a**^				**SEM^b^**	***P-*value**
	**21**	**24**	**27**	**42**		
Total SCFA, mmol/L	117^d^	88.2^e^	137^c^	133^cd^	6.45	<0.001
**SCFA, mol/100 mol**						
Acetate	60.6^c^	59.5^c^	57.1^d^	56.8^d^	0.974	<0.001
Propionate	24.5^e^	25.5^de^	27.6^cd^	29.4^c^	0.998	<0.001
Butyrate	11.1^cd^	10.6^cd^	11.5^c^	9.73^d^	0.60	0.06
Acetate:propionate ratio	2.61^c^	2.47^cd^	2.19^d^	2.03^d^	0.11	<0.01
Ruminal pH	6.14^c^	6.20^c^	5.98^d^	6.03^d^	0.065	<0.01

^a^Ruminal content was collected from each steer 6 h after feeding on days 21 (the last day of the adaptation period), 24, 27, and 42 (end of the experiment).

^b^SEM, standard error of the mean.

^cde^Means that do not have common superscript letters are different (Tukey test; *P* < 0.05).

### 3.2. Experiment 2

Feed additives affected the DMI during the adaptation period (*P* < 0.001; Table 6). Steers fed NM had greater DMI during the adaptation period compared to CON, MM, and MN (*P* ≤ 0.03; 10.4 vs. 9.73, 9.57, and 9.43 kg/day, respectively), but no differences were observed between NM and NN (*P* = 0.66) or between CON, MM, and NN (*P* ≥ 0.11). Treatments also tended to affect the DMI during the total feeding period (*P* = 0.07). Steers fed NN tended to have greater DMI compared to MM (*P* = 0.09; 10.4 vs. 9.83 kg/day) but not compared to CON, MN, and NM (*P* ≥ 0.16).

**Table 6:** Effect of feed additives on growth performance and carcass characteristics of finishing beef cattle—Exp. 2.

**Item**	**Treataments** ^**a**^					**SEM^b^**	***P*-value**
	**CON**	**MM**	**MN**	**NM**	**NN**		
Pens (steers)	8 (24)	8 (24)	8 (24)	8 (24)	8 (24)	-	-
**Growth performance**							
**Body weight, kg** ^c^							
Day 1	424	424	425	425	425	10.6	0.41
Day 21	437	442	441	442	439	10.1	0.73
Day 105	570	571	576	578	569	13.2	0.85
**Dry matter intake, kg/day**							
Adaptation period	9.73^ef^	9.57^ef^	9.43^f^	10.4^d^	10.1^de^	0.221	<0.001
Finishing period	10.1	9.90	10.3	10.4	10.5	0.260	0.15
Total feeding period	10.0^de^	9.83^e^	10.1^de^	10.4^d^	10.4^a^	0.244	0.07
**Average daily gain, kg/day**							
Adaptation period	0.590	0.819	0.761	0.821	0.701	0.150	0.78
Finishing period	1.59	1.54	1.61	1.62	1.54	0.069	0.88
Total feeding period	1.39	1.40	1.44	1.46	1.38	0.066	0.87
**Feed efficiency (gain:feed ratio)**							
Adaptation period (days 1–21)	0.060	0.084	0.080	0.079	0.070	0.015	0.79
Finishing period (days 22–105)	0.156	0.156	0.158	0.154	0.149	0.006	0.87
Total feeding period (days 1–105)	0.140	0.141	0.144	0.140	0.133	0.006	0.76
**Carcass characteristics**							
Dressing, %	57.3	58.3	58.5	58.4	57.5	0.51	0.12
Hot carcass weight, kg	328	334	336	333	332	4.00	0.70
Longissimus muscle area, cm^2^	74.8	76.7	75.3	73.2	75.9	1.69	0.64
12th-rib fat, mm	4.69^e^	6.58^d^	5.78^de^	6.05^de^	5.83^d^	0.45	0.04

^a^CON, control; no feed additive; MM, sodium monensin at 25 mg/kg DM during the adaptation and finishing periods (Rumensin, Elanco Saude Animal, São Paulo, SP, Brazil); MN, sodium monensin at 25 mg/kg DM during the adaptation period and narasin at 13 mg/kg DM (Zimprova, Elanco Saude Animal, São Paulo, SP, Brazil) during the finishing period; NM, narasin at 13 mg/kg DM during the adaptation period and sodium monensin at 25 mg/kg DM of during the finishing period; NN, narasin at 13 mg/kg DM during the adaptation and finishing periods.

^b^SEM, standard error of the mean.

^c^Shrunk body weight on days 1 and 105 (after 16 h of feed and water withdrawal). Body weight on day 21 was discounted by 4% from the full BW as ruminal fill.

^def^Means that do not have common superscript letters are different (Tukey's test; *P* < 0.05).

No other differences between treatments were observed for any of the growth performance and carcass characteristics evaluated in the present study (*P* ≥ 12), except for 12th-rib fat (*P* = 0.04). Steers fed MM had greater 12th-rib fat compared to CON (*P* = 0.02), with no differences between the other treatments (*P* ≥ 0.16).

## 4. Discussion

Feed additives such as ionophores, also known as non-nutritional ingredients, are fed to feedlot cattle to increase feed efficiency (10), decreasing the cost of production and improving the potential profit of the feedlot operation. The most common response to monensin inclusion in beef cattle diets is the increased G:F by improving or maintaining ADG and reducing DMI (10, 28, 29).

Monensin is one of the most used feed additives in finishing diets for beef cattle (7, 8). It selectively inhibits gram-positive bacteria (11), increasing the efficiency of energy metabolism (30) and nitrogen metabolism (31), and it is also used to control bloat (32), probably due to reduced feed intake variation (33) and meal size and frequency of meals (34). According to Duffield et al. (10), the average concentration of monensin in feed across 40 peer-reviewed manuscripts and 24 trial reports published from 1972 to 2003 was 28.1 mg/kg DM. Monensin decreases DMI, especially in diets with a high proportion of forage (9) due to the increased molar proportion of propionate and decreased molar proportion of acetate and butyrate (35).

Narasin is an ionophore produced by *Streptomyces aureofaciens*. It has been evaluated for pigs (36) and chicken (37), and more recently in beef cattle diets (15, 16, 18). According to these last authors, narasin fed at 13 mg/kg DM has the potential to increase ADG and improve ruminal fermentation characteristics, especially increasing the molar proportion of propionate and decreasing the molar proportion of acetate and acetate:propionate ratio, without negatively affecting DMI of beef cattle fed high-roughage diets. This characteristic would also benefit finishing cattle, especially during the adaptation period, in which finishing cattle is transitioned from high-roughage-based diets to high-concentrate diets, and the low DMI during the first 1–21 days of the adaptation period (2, 4, 6) could limit the growth performance.

In agreement with our hypothesis, steers fed narasin during the adaptation period had greater DMI compared to monensin. In Exp. 2, this difference tended to carry over the finishing period, but no differences in ADG or feed efficiency were detected between the treatments. Apparently, any difference in feed intake during the adaptation period due to feeding monensin will be compensated during the finishing phase, so no differences in the growth performance were detected at the end of the total feeding period.

The lack of treatment effect on growth performance and carcass characteristics in the present study is in agreement with Stackhouse-Lawson et al. (38) and Gouvêa et al. (39), who also did not observe differences in growth performance and carcass characteristics when monensin was fed to finishing cattle. The inclusion of 0, 22, 33, and 44 mg monensin/kg diet DM for finishing beef cattle also did not affect the DMI, rumen pH, SCFA concentrations, and H_2_S gas (40). Bell et al. (41) also did not observe differences in nutrient digestibility of beef steers receiving a forage-based diet with or without monensin. On the contrary, in a meta-analysis using 40 peer-reviewed manuscripts and 24 additional trial reports, Duffield et al. (10) concluded that monensin decreased DMI by 3% and improved G:F of finishing beef cattle by 2.5–3.5%. According to Yang and Russell (42), monensin can inhibit amino acid-fermenting ruminal bacteria, decreasing ruminal amino acid deamination and ammonia production in the rumen.

Contrary to Polizel et al. (18) and Limede et al. (19), narasin supplementation did not increase the ruminal concentration of propionate or the total concentration of SCFA in the current study. The lack of treatment effect on ruminal fermentation characteristics and nutrient digestibility agrees with the lack of treatment effect on growth performance in the current experiment.

The increase in the molar proportion of propionate and decrease in the molar proportion of acetate and butyrate throughout the days on feed is probably a result of rumen microbial change due to an increase in the amount of starch fermented in the rumen, as a combined result of dietary changes during the adaptation period and increased DMI following changes in BW.

According to Clary et al. (43), a diminished response to ionophores is observed when fat is fed in high-concentrate diets. However, according to these authors, supplementing monensin in finishing diets containing tallow (4% DM) did not affect feed efficiency but did increase the feed efficiency by 4% in diets with no tallow. According to Zinn and Borques (44), the growth performance response to monensin supplementation is reduced in fat-supplemented finishing diets. In the current study, although no supplemental fat was included in the diet, the inclusion of whole cottonseed contributed to an increase in the total fat content of the diet, which can be related to the lack of response to ionophore supplementation on the growth performance. According to Clary et al. (43), the interaction between fat and ionophores may be related to the similar effects of these two ingredients on ruminal fermentation (45) or to the decreased solubility of ionophores in lipids (43). More research is needed to better understand the nutritional and management factors that can impair monensin in finishing diets.

The lack of treatment effect on growth performance and ruminal fermentation characteristics in the current experiment is a good example of the importance of using a negative control treatment (without feed additives) when comparing different feed additives. Monensin is frequently used as the control treatment to evaluate alternative feed additives (46, 47), which makes it difficult to accurately account for the benefits (or the lack of benefits) of alternative feed additives on animal performance or ruminal fermentation characteristics.

## 5. Conclusion

Feeding narasin at 13 mg/kg DM during the adaptation period increases the dry matter intake compared to monensin at 25 mg/kg DM; however, no effects of feed additives were observed on nutrient digestibility, growth performance, or carcass characteristics of finishing cattle.

## Data availability statement

The raw data supporting the conclusions of this article will be made available by the authors, without undue reservation.

## Ethics statement

The animal study was reviewed and approved by Animal Care and Use Committee of the Luiz de Queiroz College of Agriculture, University of São Paulo.

## Author contributions

All authors listed have made a substantial, direct, and intellectual contribution to the work and approved it for publication.
